# Rubella immune status of neonates – a window towards seroprevalence among childbearing women

**DOI:** 10.1186/s12889-016-3514-y

**Published:** 2016-08-19

**Authors:** Iris Pejcic, Milica Rankovic Janevski, Aleksandra Knezevic, Djordje Jevtovic, Maja Stanojevic

**Affiliations:** 1 Institute of Neonatology, Belgrade, Serbia; 2 University of Belgrade School of Medicine, Belgrade, Serbia; 3 Infectious and Tropical Diseases University Hospital, Clinical Center Serbia, Belgrade, Serbia

**Keywords:** Rubella, Seroprevalence, IgG titer, Neonates, Mothers

## Abstract

**Background:**

When contracted in pregnancy, rubella may cause serious chronic infection of the fetus and development of Congenital Rubella Syndrome. Despite widespread application of rubella vaccination, periodical outbreaks are still being reported worldwide. The aim of this study was to determine rubella seroprevalence and antibody levels in neonates in Serbia as a proxy of maternal serostatus.

**Methods:**

ELISA based serological testing for rubella was done in 599 neonates treated at the Institute of Neonatology in Belgrade, from January 2010 to December 2011. All individuals with rubella IgG concentration ≥10 IU/ml were considered seropositive for rubella.

**Results:**

The mean age of enrolled neonates was 18 ± 6 days. The overall seroprevalence of rubella IgG antibodies among the tested neonates was 540/599(90.2 %, 95 % CI: 87.5–92.3). Seropositivity rate among sera of the neonates enrolled in 2010 was significantly higher than those collected in 2011 (*p* < 0.0001). There was no difference in average maternal age, gestational age or frequency of receiving blood products among the two study years. Significant high seropositivity rate was observed among neonates from mother aged >30 as compared to those from mothers aged <20 years (*p* = 0.02). Significant difference was also found between average IgG titers in the two study years (79 IU/mL in 2010 vs. 46 IU/mL in 2011, *p* < 0.0001).

**Conclusion:**

We report on high rubella seroprevalence among newborns in Serbia, as a proxy of rubella serostatus of childbearing aged women. Notably, declining trend of rubella antibodies toward diminishing titers suggest the importance of sustained rubella serosurvey and antenatal screening at the national level.

**Electronic supplementary material:**

The online version of this article (doi:10.1186/s12889-016-3514-y) contains supplementary material, which is available to authorized users.

## Background

Rubella virus (RV) is a single stranded positive sense RNA virus, of the family *Togaviridae,* the causative agent of an acute maculopapular rash disease. Rubella is generally presenting as mild illness, especially in children. However, when contracted in early pregnancy (8–10 weeks gestation), the risk of intrauterine transmission is up to 90 %. Maternal viremia may lead to placental infection and spread of the virus, causing a chronic infection of the fetus and development of Congenital Rubella Syndrome (CRS) [1, 2]. First rubella vaccination programs were introduced in developed countries in the late 1960s and early 1970s, and have influenced greatly the epidemiology of rubella. A number of countries have reported elimination of indigenous rubella disease. However, rubella vaccination has been introduced in differing schedules resulting in marked differences in the rubella susceptibility profile and rubella epidemiology in different countries [3]. The elimination of rubella (<1 case per million inhabitants) and control of CRS (<1 case per 100 000 live births) have been placed among the public heath priorities in Europe, with a renewed target set to 2015 [4, 5].

In Serbia, vaccination against rubella was included in the national vaccination schedule in 1994, as combined measles, mumps and rubella vaccine (MMR). Yet, the vaccine was already available in preceding years, for application on individual bases, upon parents’ request, through both the private and public sector. Until 2006, MMR vaccination was offered as single dose, for all children aged 12–15 month, while since 2006, a second dose of MMR vaccine is given to pre-school children aged 6. According to the official Serbian Ministry of Health reported data compiled by WHO and UNICEF, in the initial years of MMR vaccine introduction in Serbia, 1994 and 1995, immunization coverage was 81 and 86 %, respectively. Since, immunization coverage reached over 90 %, with periodic downfalls, such as 1999, 84 % or 2003, 87 %. For the two study years, 2010/2011, measles and rubella first dose coverage of 95 and 93 %, respectively, has been reported, however, more recently a decline below 90 % has been noted again [6]. Particularities of rubella epidemiology in Serbia both in pre vaccination era and after introduction of vaccine are only sporadically documented. Few outbreaks have been reported such as the one that occurred Belgrade in 1993, concomitant to measles outbreak, affecting mostly age groups 5–9 and 0–4 [7]. Although rubella and CRS reporting have been mandatory even before vaccine introduction epidemiological data are incomplete and discrepant, whereas surveillance data are limited, in many cases with no laboratory confirmation [8]. However, based on the WHO data, the number of reported rubella cases in Serbia in the last decade is gradually declining. Estimated rubella incidence in Serbia, based on the annual number of reported cases, dropped from 31.3 per 100.000 in 2000 to 0.2 per 100.000 in 2010 [9]. No cases of CRS were reported in the last decade, however, in view of no active surveillance for rubella, as qualified by WHO, these data might subject to under-reporting [10]. Apart from serosurvey data which reported seroprevalence of >97 % among women of reproductive age in 90’s, no recent data exist about rubella seroprevalence in any target population in Serbia [11]. Hence, in spite of solid MMR uptake, in the view of changing vaccination strategies and possible gaps in surveillance it is important to assess rubella serostatus among target groups such as pregnant women.

Since it is well known that transplacental transport allows transfer of maternal IgG antibodies to offspring across the placenta, newborn antibody levels correlate to maternal antibody status and concentration [12]. This study was undertaken to assess rubella seroprevalence and antibody levels in preterm and full-term neonates treated at the Institute of Neonatology in Belgrade, Serbia, in 2010–2011, as a proxy for maternal antibody status and concentration.

## Methods

Serological testing for rubella was done within blood screening for TORCH agents (Toxoplasma gondii, Rubella virus, Cytomegalovirus, Herpes simplex virus) of neonates treated at the Institute of Neonatology in Belgrade, from January 2010 to December 2011. Institute of Neonatology in Belgrade, Serbia is a specialized tertiary pediatric healthcare hospital, serving as the national reference center for neonatal care. This institution is treating around 1000 neonatal patients yearly. All treated patients are referred from other maternity units all over the country. Patients were included upon broad clinical criteria suggesting possible intrauterine infection. Indications for rubella serology testing were not limited to case definition of CRS, but included the presence of diverse inflammatory, metabolic and congenital anomalies, central nervous system anomalies, intrauterine growth restriction. All eligible neonates were recruited upon parents’ consent, and blood samples were collected inpatient, during initial hospitalization upon birth, the length of which depended on clinical course and outcome. The outliers were excluded from statistical analysis. We had no access to the information about maternal rubella vaccination status. The study was conducted under the approval of the Ethical committee of the Institute of Neonatology.

Rubella testing was carried out using commercial Enzyme Linked Immunosorbent Assay (ELISA) tests for anti-rubella virus IgM and IgG (Anti-Rubella Virus ELISA, Euroimmun, Luebeck, Germany), according to manufacturer’s instructions. For the used anti-rubella virus IgG test the manufacturer reported test specificity and sensitivity of 100 and 99.6 %, respectively, whereas reported specificity and sensitivity for the IgM test were 98.6 and 96.4 %. The calibrators used for the qualitative IgG assay were 10 IU/ml, 50 IU/ml and 200 IU/ml. All individuals with rubella IgG concentration ≥10 IU/ml were considered seropositive and those with concentrations below that threshold were considered seronegative for rubella. Opposite to early seroepidemiological studies based on classical serological reactions (HI, radial immunodiffusion) that considered 15 IU/ml as the minimum rubella immune titre [13, 14], we used 10 IU/ml as the positive/negative cut-off for rubella IgG antibody detection and evidence of protection, according to more recent epidemiological studies and widespread use of more sensitive techniques such as ELISA [15, 16]. All samples that tested positive for anti-rubella virus IgM antibodies were repeatedly tested using the Enzygnost Anti-Rubella Virus/IgM (Siemens Healthcare Diagnostics, Marburg, Germany), as per manufacturer’s instructions.

Obtained results were processed using standard statistical analysis. All analyses were performed using an electronic database organized in the SPSS (version 11.5) statistical package Prevalence values were calculated with a 95 % Wilson score confidence interval based on a binomial distribution. Categorical data were compared using the chi-square test or Fisher’s exact test, while continuous data were investigated by means of a *t*-test, Mann–Whitney *U* test and ANOVA.

## Results

A total of 599 neonates were enrolled during the study period whereby 405 and 194 were enrolled in 2010 and 2011 respectively. Mean newborn’s gestational age (GA) was 32.7 ± 3.8 weeks, whereas mean age at which blood sample was drawn for testing was 18.6 ± 6 days (range 1–148 days). Mean maternal age was 30.2 ± 6 years (range 15–48 years) (Table 1). The most prevalent indications for testing, in two thirds of the samples (374/599), were intrauterine growth restriction (IUGR) and/or suspicion of central nervous system disorder. No significant difference was identified between the two study groups (2010 vs. 2011) regarding maternal/infant age, GA, age at sampling or indications for testing.

**Table 1:** Serological findings and general clinical and demographic data according to study year: seropositive = >10 IU/mL; low titer = 10–15 IU/mL; GA – gestational age in weeks; age sampling – mean age of sampling in days; BP % – percentage of subjects havinh received blood/blood products; maternal age – mean age of mothers; *SD* standard deviation, *CI* confidence interval

	Total	2010	2011	*p*
*Serological findings*				
Seropositive (%)	540/599 (90.2 %)	380/406 (93.6 %)	160/193 (82.9 %)	<0.0001
Mean IgG titer (SD)	71 IU/mL (62)	79 IU/mL (62)	46 IU/mL (57)	<0.0001
% low titer IgG (95 % CI)	5 (3.5–7)	4.9 (3.2–7.5)	5.2 (2.8–9.2)	-- ^a^
*Clinical and demographic data*				
Male No (%)	311 (51.4 %)	201 (49.6 %)	104 (53.6 %)	--
Female No (%)	294 (48.6 %)	204 (50.4 %)	90 (46.4 %)	--
GA (SD)	32.7 (3.8)	32.5 (3.9)	32.9 (3.5)	--
Mean age of neonates (SD)	18.6 (22.1)	20.1 (24.3)	16.9 (19.2)	--
BP % (95%CI)	34.7 (31.0–38.6)	35.1 (30.6–39.8)	32.6 (26.4–39.5)	--
Maternal age in years (range)	30.2 (15–48)	30.1 (16–48)	30.3 (15–45)	--

^a^indicates no statistically significant difference

**Fig. 1:**
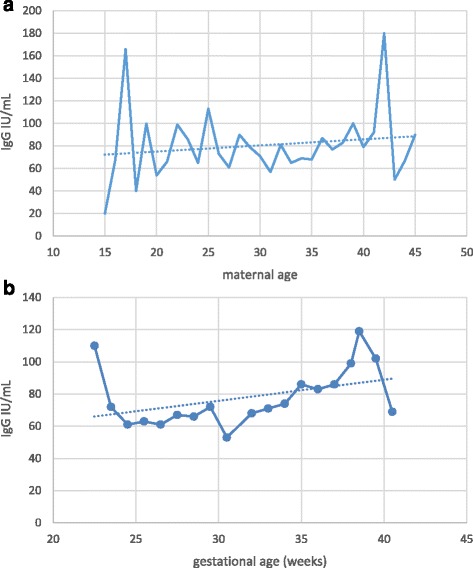
**a** Anti-rubella IgG titer in newborns in relation to maternal age. **b** Anti-rubella IgG titer in newborns in relation to gestational age

Serological testing revealed that 540/599 tested neonates were positive for IgG antibodies to rubella, corresponding to the overall seropositivity rate of 90.2 % (95 % CI: 87.5–92.3 %) (Table 1). Seropositivity rate among samples from 2010 was 93.6 % (95 % CI: 90.8–95.6 %), whereas in samples from 2011 it was found to be 82.9 % (95 % CI: 76.9–87.6 %), the difference yielding high statistical significance (*p* < 0.0001). There was no difference in average maternal age, gestational age or frequency of receiving blood products among the two study groups. Excluding from the analysis samples of patients having received blood/blood products did not influence the prevalence rate. The highest seropositivity rate was found among newborns of mothers aged over 30 years, 93.6 % (95 % CI: 89.9–96.0 %), while seroprevalence was the lowest among newborns of mothers younger than 20, 78.3 % (95 % CI: 58.1–90.3 %), with significant increase of seroprevalence rate with age, *p* = 0.02 (Fig. 1, Table 2). There was no significant difference in seroprevalence with regards to infant’s gestational age (Fig. 1, Table 2).

**Table 2:** Rubella seroprevalence and titer in relation to maternal age and gestational age of newborns

Maternal age	<20	20 - 30	>30	*p*
% seropositive (95 % CI)	78.3 (58.1–90.3)	89.3 (85.0–92.4)	93.6 (89.9–96.0)	0.0182^a^
Mean IgG titer (SD)	60 IU/mL (67)	75 IU/mL (65)	72 IU/mL (62)	--^b^
GA (weeks)	<32	>32		
% seropositive (95 % CI)	90.1 (86.7–92.8)	90.1 (85.6–93.3)		--
Mean IgG titer (SD)	59.5 IU/mL (53)	78 IU/mL (66)		*p* < 0.0001

^a^chi-square test for trend

^b^indicates no statistically significant difference

Overall, mean anti-rubella IgG titer was found to be 71 IU/mL (95 % CI: 66.0 to 75.9 IU/ml); significant difference was found between average IgG titers in the two study years: 79 IU/mL in 2010 (95 % CI: 72.9 to 85.0 IU/ml) vs. 46 IU/mL in 2011 (95 % CI, 37.9 to 54.1 IU/ml), *p* < 0.0001. Excluding from the analysis samples of patients having received blood/blood products did not influence the mean anti-rubella IgG titer. Among newborns of gestational age (GA) less than 32 weeks, mean anti-rubella IgG titer was found to be 59.5 IU/mL (95 % CI, 52.6 to 62.4 IU/ml), this was significantly lower compared to the average titer in newborns of GA > 32 weeks of 78 IU/mL (95 % CI, 70.9 to 85.0 IU/ml), *p* < 0.0001.

In five out of 599 samples the first ELISA testing revealed positive finding of anti-rubella virus IgM antibodies, whereupon two samples were confirmed to be rubella virus IgM positive on confirmatory analysis. Further PCR testing of these 2 samples and serological and clinical follow-up did not confirm CRS diagnosis (data not shown).

## Discussion

Here, we present the first study of the prevalence and titer of anti-rubella antibodies among preterm and full-term newborns in Serbia. We report on high rubella seroprevalence among newborns in Serbia, as a proxy of rubella seroprevalence among their mothers - women of reproductive age. Of note, high seroprevalence was found, albeit with a decline in titer in the study period.

Widespread use of rubella vaccine has dramatically reduced the disease burden around the globe. In 1996, the first global survey on the use of rubella vaccines found that it was administered in 83 countries, including Serbia [17]. By 2010, when our study was initiated, that number reached 130. In Europe, 74 % of countries had introduced rubella vaccination in routine schedule by 1996, whereas by 2010 this was the case in all the countries of the WHO European region [18]. Nonetheless, periodical rubella outbreaks are still being reported worldwide. Despite rubella vaccination being present in Japan since mid-seventies of the last century, in 2012/2013, a large rubella outbreak emerged in that country, in particular in Tokyo region [19]. Nearly a quarter of these cases, 23 %, were in females. During this outbreak, since October 2012, 10 CRS cases have been reported in Japan [19]. Increased risk for CRS has been noted in some European countries also: in 2013, Poland reported over 20,000 rubella cases (55.2 per 100,000 inhabitants) [20]. Recently, rubella outbreaks have also been reported in some Balkans countries, sharing borders with Serbia. According to WHO vaccine-preventable diseases monitoring data, in 2011/2012 an outbreak involving nearly 25,000 rubella cases was reported in Romania, with 126 CRS cases reported in the period 2012–2014 [21]. Over 2000 cases were reported in the north-west Romanian region of Salaj [22]. The incidence of rubella in Salaj reached 763 cases per 100,000 populations, the highest one being among high school teenagers, both girls and boys, aged between 15 and 19 years. Vaccination coverage among the reported cases was low, 2.1 % of the total number of cases were vaccinated with at least one dose of rubella-containing vaccine [22]. In 2009 an outbreak of rubella was reported in Bosnia and Herzegovina, involving several hundred cases and affecting mainly unvaccinated or partially vaccinated 16–17 year-old school children [23].

In our study we assess rubella seroprevalence among women of reproductive age through serosurvey among newborn infants. Placental transfer of maternal IgG antibodies to the fetus is an important mechanism that provides protection to the infant; hence seroprevalence among newborn infants reflects the one of their mothers. In the neonatal population under study we found an overall seropositivity rate of 90.2 % that reflects the seroprevalence among women of childbearing age. Comparison of rubella seroepidemiology in 16 European countries and Australia in the period 1996–2004 found an overall seroprevalence ranged from 42.9 to 99.1 % [24]. However, in the adult population, in particular among women of childbearing age, rubella seroprevalence was similar to the one we found, ranging from 86.6–98.6 %. This study included countries with diverse vaccine practice and coverage: from no rubella vaccination in the schedule at time of analysis to single or double dose schedule. A recent study in The Netherlands found an overall nationwide seroprevalence of 95 %, after more than three decades of high vaccination coverage [25]. Herd immunity of about 80 % is considered protective and high vaccine coverage is expected to account for adequate seroprevalence [16]. According to the official Serbian Ministry of Health reported data compiled by WHO and UNICEF, for the two study years, 2010/2011, measles and rubella first dose coverage of 95 and 93 %, respectively, has been reported, however, more recently a decline below 90 % has been noted again [6]. Hence, in spite of solid vaccine coverage, unreached pockets of population have been identified, such as Roma people from unofficial settlements, largely out of scope of the public health system [26]. Suboptimal vaccination coverage has been shown to induce an increase in congenital rubella syndrome occurrence, due to a decreased circulation of the virus and an accumulation of susceptible adult females, as documented previously in some European countries [27–29]. Several recent reports describe declining rubella seroprevalence and waning rubella immunity at the population level, in particular among women of childbearing age, in decades following introduction of rubella vaccination [30, 31]. Our results of significantly lower seroprevalence and titer may also indicate diminishing rubella seroprevalence in the second study period, although the analyzed timeframe is narrow, encompassing only 2 consecutive years. We had no access to the information about maternal rubella vaccination status. However, knowing that rubella vaccine was included into the national vaccination calendar in 1994, we may hence assume that cohorts of mothers born before 1992 had not been vaccinated. Since the study period was less than 20 years post rubella vaccine introduction and the average maternal age in the study was around 30 (range 15–48), we may conclude that large majority of seropositive infants were born to mothers who acquired immunity after natural rubella infection, the finding which is supported by previous studies [32, 33]. This may be due to the difference in acquiring exposure to RV antigens. Seroepidemiological studies have demonstrated that antibody response following natural rubella infection is higher and more durable than the one induced by immunization [34]. After rubella vaccination antibody avidity rises in much slower pace, with high avidity antibodies detected in less than 10 % of vaccinees five months post vaccine administration, in 20–40 % at 5–9 months, and in 50 % at 10–12 months. In approximately 30 % of vaccinees, avidity will remain at moderate levels for many years [16]. Titers of maternal antibodies to some vaccine preventable diseases have been shown to be lower and more rapid to decline in children from vaccinated mothers [35].

Possible limitations to our study refer to the number of included neonates and the fact that majority of tested newborns were preterm. Regarding the study size, in total and in two consecutive years, we found no difference in average maternal age, gestational age or frequency of receiving blood products among the two study groups; hence, we believe that these factors might not fully explain the differences observed. On the other hand, regarding the impact of preterm delivery to the obtained serological findings: there is evidence that placental IgG transfer depends on multiple factors, such as maternal levels of total and specific IgG antibodies, gestational age, placental integrity, IgG subclass etc. [36]. Most of the antibodies are passed in the third trimester of pregnancy. Fetal IgG rises from approximately 10 % of the maternal concentration at 17–22 weeks of gestation to 50 % at 28–32 weeks of gestation [37]. At term, fetal IgG typically somewhat exceeds maternal levels. Nevertheless, although studies of rubella specific antibodies have reported lower antibody titers in preterm compared to term infants, similar to our findings previous studies have also shown that majority (50–90 %) of preterm infants did possess a protective rubella antibody titer [38, 39]. In addition, the proportion of subjects with low level anti-rubella IgG titer (10–15 IU/mL) was similar in both study periods. Our finding of lower anti-rubella IgG titer among newborns of GA less than 32 weeks compared to those of GA over 32 weeks is in accordance to previous reports and in line with expected dynamics of transplacental antibody transfer [39, 40]. Importantly, follow-up studies have documented that IgG titers of preterm infants decrease earlier in life below protective antibody titers than term infants [39]. Consequently, lower titers of transplacentally acquired IgG in preterm than in term infants, pose preterm infants at higher risk of vaccine preventable diseases early in life. Our findings of lower anti-rubella seroprevalence and titer in younger cohorts are concordant to documents of diminishing herd immunity among women of reproductive age and raise the question of possible widening of the window of susceptibility to infectious disease in the first year of life [41]. Ongoing debate on the need to reassess vaccination schedule is of particular importance for premature, but also for infants born at term [42].

## Conclusion

In conclusion, we report on high rubella seroprevalence among newborns in Serbia, as a proxy of rubella seroprevalence among childbearing women. Notably, a tendency towards diminishing anti-rubella IgG titer was found in the study period, implying continued need for rubella serosurvey and antenatal screening. High immunity against rubella, especially in pregnant women, is prerequisite for achieving the goal set by WHO to eliminate endemic rubella and CRS in the European region, highlighting the importance of seroepidemiological studies to monitor epidemiological status and vaccination program at the national level.

## Additional file

Additional file 1: Serology results with basic demographic and clinical data for the studied population. (XLSX 106 kb)
